# High Grade Glioma Mimicking Voltage Gated Potassium Channel Complex Associated Antibody Limbic Encephalitis

**DOI:** 10.1155/2014/458790

**Published:** 2014-01-12

**Authors:** Dilan Athauda, R. S. Delamont, E. De Pablo-Fernandez

**Affiliations:** ^1^Neurology Department, Darent Valley Hospital, Darenth Wood Road, Dartford & Gravesham NHS Trust, Dartford DA2 8DA, UK; ^2^Neurology Department, Kings College Hospital, London SE5 3RS, UK

## Abstract

Though raised titres of voltage gated potassium channel (VGKC) complex antibodies have been occasionally associated with extracranial tumours, mainly presenting as Morvan's Syndrome or neuromyotonia, they have not yet been reported to be associated with an intracranial malignancy. This is especially important as misdiagnosis of these conditions and delay of the appropriate treatment can have important prognostic implications. We describe a patient with a high grade glioma presenting with clinical, radiological, and serological features consistent with the diagnosis of VGKC antibody associated limbic encephalitis (LE). This is the first association between a primary brain tumour and high titre of VGKC complex antibodies. Clinicoradiological progression despite effective immunosuppressive treatment should prompt clinicians to look for alternative diagnoses. Further studies to elucidate a possible association between VGKC complex and other surface antigen antibodies with primary brain tumours should be carried out.

## 1. Introduction

VGKC complex antibody associated LE is considered a nonparaneoplastic, immunoresponsive condition typically presenting subacutely with memory loss, confusion, seizures, and behavioural changes (though visual hallucinations and psychosis have also been reported) [1]. Approximately 60% of patients have high signal changes unilaterally or bilaterally in the medial temporal lobes on FLAIR and T2 weighted MR imaging [1]. Serum hyponatremia, another diagnostically useful feature, is present in around 60% of patients [2]. The diagnosis is usually made with a combination of clinicoradiological findings and the presence of high titre VGKC complex antibodies (normal reference range is <100 picomolar (pM)). Though it is uncertain at what level a titre of antibody becomes significant, previous studies have suggested titres greater than 400 pM as “high” and levels above this seem to be more associated with CNS disease [1, 2]. Though the phenotype of VGKC complex antibody associated LE is continually expanding, other important conditions can often mimic the initial presentation and alternative diagnoses should be considered in atypical cases. Intracranial malignancy is an important differential diagnosis and can present with similar symptoms; however, there have been no reports of elevated levels of VGKC complex antibodies being found in association with an intracranial malignancy. We report a case of a patient presenting initially with characteristic features of VGKC complex associated LE with elevated levels of VGKC antibodies in his serum who, despite treatment with immunosuppression, clinically deteriorated and was subsequently discovered to have a high grade glioma.

## 2. Case Presentation

An 86-year-old man complaining of 1 week of lethargy and irritability was found unconscious at home and taken to the emergency department whereupon he had two generalised tonic clonic (GTC) seizures. He had no past medical history and was on no medication. There was no history of recent illness, cognitive decline or travel.

On examination he had a temperature of 38°C and had a GCS of 10. Though his consciousness improved over the next few hours he remained confused and verbally and physically aggressive. Repeated neurological examination was normal. He was started on ceftazidime, amoxicillin, and acyclovir for a possible CNS infection.

**Figure 1:**
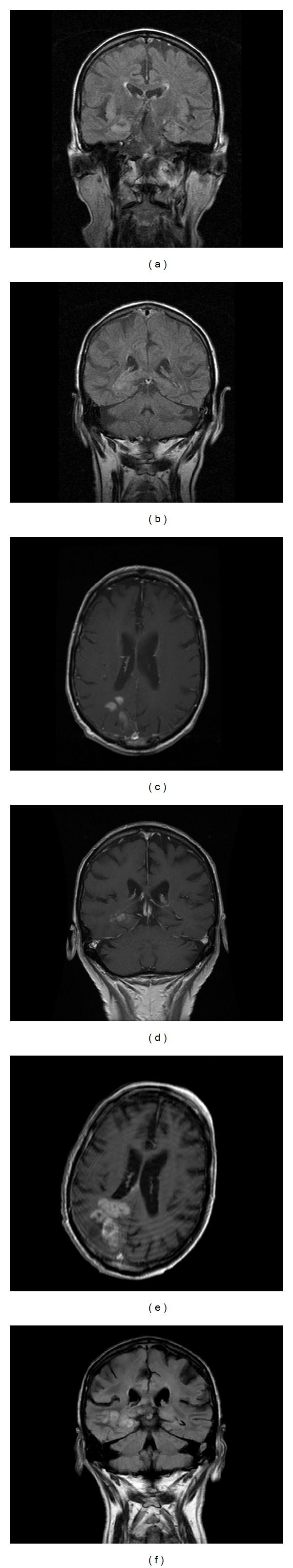
(a, b) Coronal T2 weighted FLAIR brain MRI showing high signal changes involving medial temporal areas. (c, d) T1 weighted MRI 10 weeks later showing enhancement of the previous lesions after gadolinium administration with extension posterior to the occipital lobe. (e, f) T1 weighted brain MRI showing rapid progression of the initial lesion with marked mass effect, areas of necrosis, and heterogeneous contrast enhancement consistent with a high grade glioma.

Extensive blood tests including full blood count, electrolytes, liver and thyroid function tests, CRP, ESR, ANA, ANCA, anticardiolipin antibodies, vitamin B12, folate, ACE, protein electrophoresis, *Borrelia*, and HIV serologies were negative or normal. His initial brain CT scan without contrast was normal and CSF analysis revealed a white cell count of 35 (30% lymphocytes), with normal protein and glucose, culture and viral PCR. MRI brain showed high signal changes on T2 weighted images involving the right insula, parahippocampal gyrus, hippocampus, and splenium of the corpus callosum suggestive of limbic encephalitis (Figures 1(a) and 1(b)). A paraneoplastic screen (for anti-Yo, anti-Hu, and anti-Ri antibodies) was negative and CT chest, abdomen, and pelvis did not identify any occult malignancy. His confusion and behavioural abnormalities resolved a few days after admission and he was discharged after completing 14 days of treatment with intravenous acyclovir. An extended autoimmune screen was sent including voltage gated potassium channel (VGKC) complex antibodies, N-methyl-D-aspartate receptor (NMDAr), and glutamic acid decarboxylase (GAD) antibodies.

Unfortunately, he was readmitted 15 days later with fluctuating disorientation, aggressiveness, and complex visual hallucinations. Neurological examination was unremarkable including cognitive assessment (Mini-Mental State Examination score was 30/30). His extended immune screen returned which was negative for NMDAr and GAD antibodies, but he had a raised VGKC complex antibody titre of 437 picomolar (pM). Repeat MRI brain scan 5 weeks after initial presentation showed persistent high T2 signal changes of the right medial temporal lobe structures previously described with new involvement of the right precuneus (Figures 1(c) and 1(d)). A diagnosis of VGKC complex antibody limbic encephalitis (LE) was made. The patient declined treatment with plasmapheresis or intravenous immunoglobulins and was started on oral prednisolone 40 mg a day. He improved and was discharged. Repeat serum testing sent for VGKC antibodies 3 weeks after starting the steroids was now undetectable.

He presented 25 days later with 3 generalised tonic clonic seizures and gradual progressive cognitive decline. On examination, he was disorientated, had mild left sided hemiparesis, and was neglecting the left side. He underwent a course of plasmapheresis for 5 days, but a repeat MRI brain scan following the plasmapheresis showed aggressive progression of the previous lesions, with new nodular foci within the medial aspect of the right temporoparietal lobes with mass effect, heterogeneous contrast enhancement, and areas of necrosis more in keeping with a high grade glioma than LE (Figures 1(e) and 1(f)).

Following review by a neurooncology multidisciplinary team he was offered supportive care only. He subsequently had a gradual deterioration of his condition with progressive cognitive decline and general physical decline and occasional generalized tonic seizures despite antiepileptic treatment.

## 3. Discussion 

Our patient initially illustrated many of the classic clinical, radiological, and serological features of VGKC antibody complex LE: he had well-recognised symptoms of seizures, amnesia, and personality change. He had unilateral high signal changes in his medial temporal lobe on MRI T2 weighted imaging which is usually observed in such cases. We also detected elevated levels of VGKC antibody in his serum of 407 pM—usually undetectable. The suggested treatment for VGKC complex associated LE is early aggressive immunotherapies with a combination of high dose steroids, plasma exchange, or intravenous immunoglobulins, with a concurrent rapid fall of the antibody level with effective therapy [3]. Typically prognosis is generally good though radiological changes may take longer to improve, with subsequent persistent temporomesial atrophy as a common finding [1].

Despite the subsequent immunosuppressive therapies, the patient developed progressive cognitive decline, visual hallucinations, and left sided hemiparesis, with spreading of the MRI changes involving new areas including the medial parietal lobe and nodular enhancement—which is atypical for VGKC associated LE. Though no histological confirmation is available, repeat brain MRI 8 months after the initial presentation showed radiological findings in keeping with an aggressive, high grade glioma.

The spectrum of conditions associated with VGKC complex antibodies is in continuous expansion and raised titres of VGKC complex antibodies have also been reported in other clinical syndromes including Morvan's Syndrome, neuromyotonia, and faciobrachial dystonic seizures [1]. Though neuromyotonia and Morvan's Syndrome can be associated with tumours, particularly thymomas, they are uncommon in VGKC complex antibody LE and it is thought to be a nonparaneoplastic condition.

A study to identify possible neurological and oncological associations in patients with raised VGKC antibodies found that 33% of patients identified with raised VGKC antibodies had active tumours, mostly lung carcinomas, but also 3 haematological malignancies and 1 thymoma [4], though no distinction between different clinical syndromes was made.

However, a more recent study that identified the clinical phenotype and tumour association of 96 patients with VGKC antibody titres >400 pM found that the majority—66%—had LE, and of these, none were associated with underlying tumours. Of the six patients identified in the study with active tumours all had either Morvan's Syndrome or neuromyotonia, 5 had thymomas and one patient had endometrial adenocarcinoma [5].

To our knowledge, no intracranial malignancy, either primary or metastases, has been associated with raised VGKC antibodies and this is the first case reporting this association. This is extremely important as misdiagnosis of these conditions and delay of the appropriate treatment could have important prognostic implications.

It is impossible to ascertain that if an early diagnosis in our case could have made an impact on his prognosis though, as once the diagnosis was made, the patient was only suitable for supportive care. This case illustrates that clinicians treating patients with a presumed diagnosis of VGKC complex antibody LE must maintain a high index of suspicion, even in the presence of high levels of antibodies, and should consider appropriate neuroimaging and alternative diagnoses if the patient fails to respond to effective immunotherapy. It also highlights the difficulty of diagnosing gliomas in the early stages despite MR imaging. Further studies to investigate a possible association between VGKC complex and other surface antigen antibodies with primary brain tumours should be carried out in order to better elucidate their role in these conditions.
